# Life-threatening hemoptysis accompanied by internal thoracic artery aneurysms in a 28-year-old perinatal woman: a case report

**DOI:** 10.1186/s12890-021-01538-y

**Published:** 2021-05-19

**Authors:** Yujun Li, Yuyao Wang, Zhike Liang, Chuzhi Pan, Xiaomei Huang, Zexun Mo, Guodong Chen, Dongliang Zhu, Ziwen Zhao, Shuquan Wei

**Affiliations:** 1 Department of Pulmonary and Critical Care Medicine, Guangzhou First People’s Hospital, School of Medicine, South China University of Technology, 1 Panfu Road, Yuexiu District, Guangzhou, 510180 Guangdong China; 2 Department of Medicine, Danbury Hospital, Danbury, CT USA; 3 Department of Hepatobiliary Surgery, The Third Affiliated Hospital of Sun Yat-sen University, Tian He Road, Guangzhou, Guangdong China; 4 Department of Interventional Radiology, Guangzhou First People’s Hospital, School of Medicine, South China University of Technology, Panfu Road, Guangzhou, Guangdong China; 5 Department of Interventional Operation Center, Guangzhou First People’s Hospital, School of Medicine, South China University of Technology, Panfu Road, Guangzhou, Guangdong China

**Keywords:** Life-threatening hemoptysis, Internal thoracic artery aneurysms, Perinatal, Bronchial artery embolization, Case report

## Abstract

**Background:**

Life-threatening hemoptysis presents an immediate diagnostic and therapeutic challenge, especially during the perinatal period.

**Case presentation:**

A 28-year-old perinatal woman with no significant past medical or surgical history presented with repeating hemoptysis and respiratory failure. Computed tomography revealed a 2.1 × 3.2  cm^2^ inhomogeneous tumorous lesion in the right superior mediastinum and a right main bronchus obstruction along with atelectasis of the right lung. Bronchoscopy showed a tumorous protrusion blocking the right main bronchus with active hemorrhage, and malignancy was suspected. Bronchial artery embolization (BAE) was performed to control the bleeding. The arteriogram revealed tortuosity, dilation and hypertrophy of the right bronchial arteries and aneurysms of the internal thoracic artery (ITA). The bleeding completely stopped after BAE. Bronchoscopy was performed again to remove residual blood clots. The patient recovered soon after the procedure and was discharged.

**Conclusions:**

Life-threatening hemoptysis concomitant with ITA aneurysms, which may have a misleading clinical diagnosis and treatment options, has not been reported previously in perinatal women. BAE could be used as a first-line treatment irrespective of the underlying causes.

## Background

Hemoptysis is considered life threatening if, regardless of the degree, it is associated with respiratory failure from airway obstruction or hypotension [1]. Bronchiectasis, tuberculosis, and malignancies are the top three causes of this condition [1, 2]. However, the origin of the bleeding and the underlying etiology are often not immediately apparent, which may present an immediate diagnostic and therapeutic challenge. Here, we present a perinatal patient who had life-threatening hemoptysis concomitant with internal thoracic artery (ITA) aneurysms and pulmonary atelectasis, an extremely rare event for life-threatening hemoptysis management.

## Case presentation

A 28-year-old G3P2A1 woman with no significant past medical or surgical history was admitted on postpartum day 4 and presented with repeating hemoptysis and increasing dyspnea. She coughed up 10–20 mL fresh blood for 4 days and complained of dyspnea that increased on exertion and was relieved by rest after spontaneous vaginal delivery. Her pregnancy and delivery were uneventful, with a healthy female child at 38 weeks and 1 day. Her first pregnancy was miscarried at 2 months, and her second pregnancy went well and gave birth to a girl by vaginal delivery. The girl is 3 years old and doing well. Social and family history were unremarkable.

**Fig. 1:**
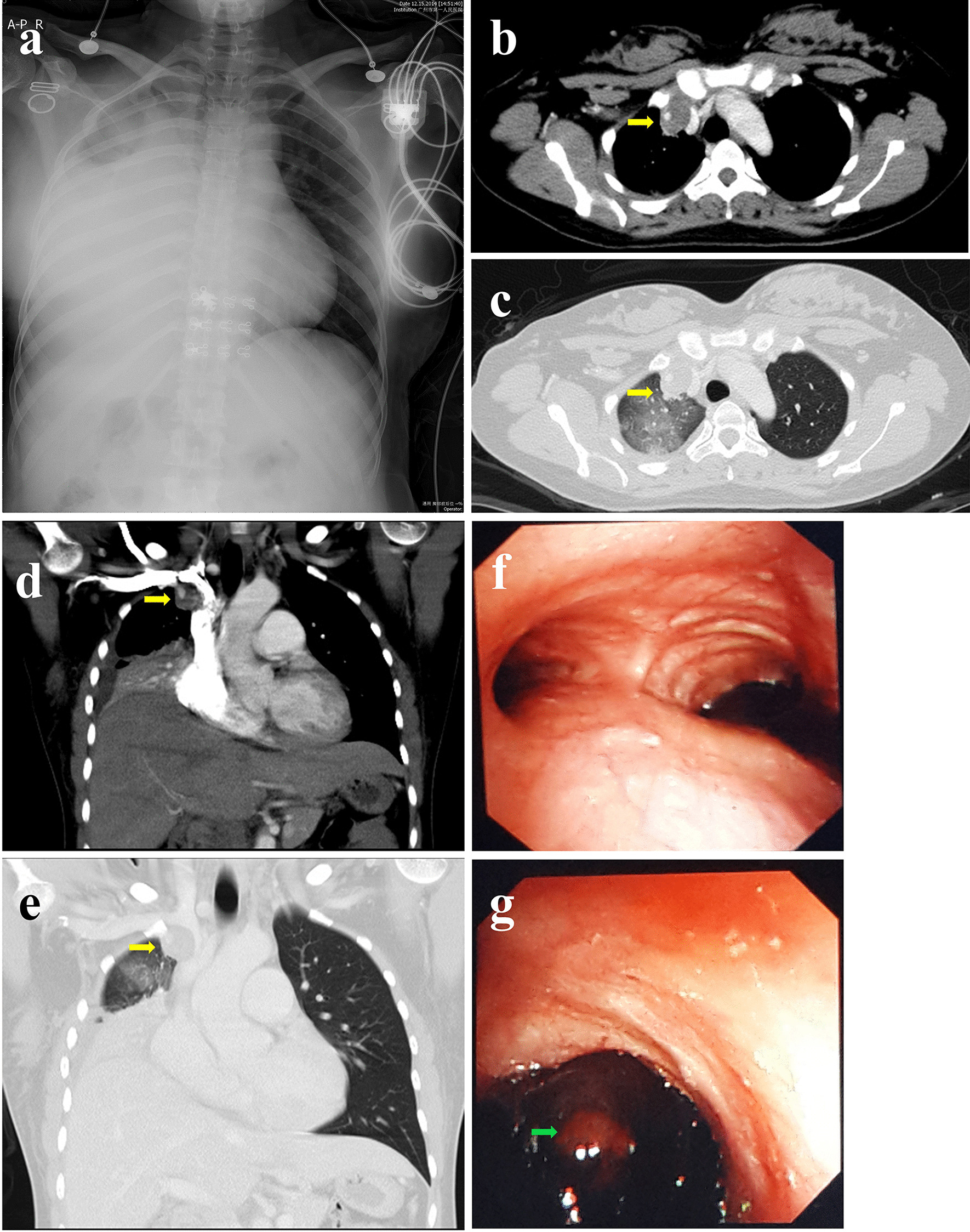
Chest radiograph indicating atelectasis of the right middle and lower lung (**a**). Computed tomography shows a 2.1 × 3.2 cm^2^ inhomogeneous tumorous lesion (yellow arrow) in the right superior mediastinum on axial views (**b** mediastinal windows; **c** lung windows) and coronal views (**d** mediastinal windows; **e** lung windows). Bronchoscopy shows tracheal carina (**f**) and right-side mainstem bronchus (**g**) blocked by a tumorous protrusion (green arrow) with active hemorrhage

**Fig. 2:**
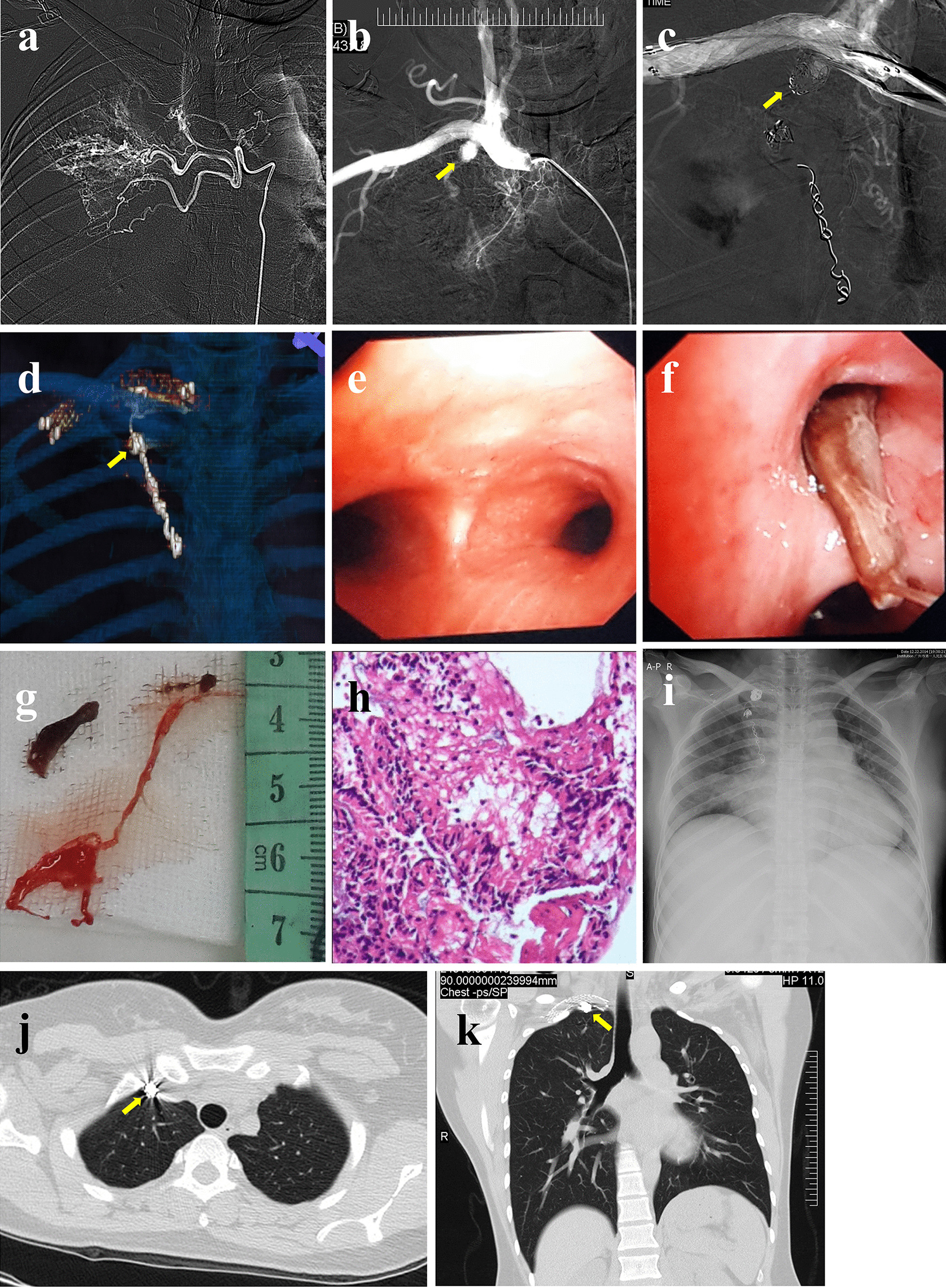
Arteriogram revealing the tortuosity, dilation and hypertrophy of the right bronchial arteries (**a**). Aneurysms (yellow arrow) of the internal thoracic artery before (**b**) and after (**c**) metal coil embolization. Three-dimensional image shows aneurysms embolized by a metal coil (yellow arrow) (**d**). Bronchoscopy was used to remove blood clots (**g**), as confirmed by pathological examination (**h**), that were blocking the right-sided mainstem bronchus (**e**) and upper lobe bronchus (**f**). Chest radiograph indicates reexpansion of the right lung after removal of the clots (**i**). Follow-up CT shows the metal coil (yellow arrow) and the right lung returned to normal (**j** axial views; **k** coronal views)

On examination, the patient was alert and oriented, with temperature 37.5 °C, heart rate 125, respiratory rate 28, and oxygen saturation 88% on room air. Physical examination was significant for tachycardia; otherwise, the patient presented with a regular rhythm, dullness to percussion, decreased tactile fremitus, and no breath sounds to the right side. The skin showed no sign of petechiae and ecchymosis. Laboratory tests revealed fibrinogen (FIB) 5.82 g/L, thrombin time (TT) 11.5 s, prethrombin time ratio (PTR) 0.86, activated partial thromboplastin time (APTT) 26 s, prothrombin time (PT) 11 s, international normalized ratio (INR) of PT 0.88; D-dimer 620 μg/L; white blood cell count 11.2 × 10^9^/L, Platelet count 223 × 10^9^/L, Hemoglobin 88 g/L, Red blood cell count 4.05 × 10^12^/L; mean corpuscular volume (MCV) 78.8 fL, mean corpuscular hemoglobin (MCH) 21.7 pg, and mean corpuscular-hemoglobin concentration (MCHC) 276.0 g/L. The absolute counts and percentages of other blood cells were within the normal ranges. Arterial blood gas analysis on 5 L nasal cannula showed pH 7.385; pO_2_ 78 mmHg; pCO_2_ 32.5 mmHg, HCO_3_ 22 mmol/L; O_2_ saturation 95.8 %, PaO_2_/FiO_2_ ratio 190. The rest of the laboratory results, including the comprehensive metabolic panel, inflammation markers, and pro-B-type natriuretic peptide (pro-BNP), were all within normal limits. Autoimmune and vasculitis panels were negative. Complement levels and immunoglobin levels were all unremarkable. Chest radiographs suggested right lung atelectasis (Fig. 1a). Although D-dimer and FIB were mildly elevated, the patient never complained of any epistaxis or bleeding gums; combined with a normal platelet count and the absence of petechiae and ecchymosis, hemoptysis caused by coagulation dysfunction was not supported. CT was further performed to determine the causes of bleeding, revealing a 2.1 × 3.2  cm^2^ inhomogeneous tumorous lesion in the right superior mediastinum and a right main bronchus obstruction along with right upper lobe obstructive pneumonia and atelectasis of the right lung, particularly the right middle and lower lobes (Fig. 1b–e). No evidence of pulmonary embolism. Flexible bronchoscopy showed a tumorous protrusion blocking the right main bronchus with active hemorrhage (Fig. 1f, g). Malignancy was suspected. Although the cause of the bleeding was unclear, biopsy was not performed because the irritation produced by the biopsy procedure may have caused rupture of unidentified aneurysms or repeating, massive bleeding. Interventional angiography and bronchial artery embolization (BAE) were performed instead. The arteriogram revealed tortuosity, dilation and hypertrophy of the right bronchial arteries (Fig. 2a). Angiography showed aneurysms of the internal thoracic artery (Fig. 2b). The right bronchial arteries were embolized with 500 μm polyvinyl alcohol particles and gelatin sponges. The aneurysms of the internal thoracic artery were embolized with a metal coil (Fig. 2c, d). A stent was placed in the opening of the right internal thoracic portion of the right subclavian artery. The bleeding completely stopped after BAE. Bronchoscopy was performed again to remove residual materials (Fig. 2e–g). Pathology confirmed these were blood clots (Fig. 2h). The patient recovered soon after the procedure (Fig. 2i) and received regular outpatient follow-up (Fig. 2j, k).

## Discussion and conclusions

Life-threatening hemoptysis is a medical emergency with high mortality presenting with several difficult diagnostic and therapeutic challenges. Identification of the underlying causes is important. A prospective study with 606 patients was performed to investigate the etiology of hemoptysis and revealed that compared with CT scans (77.3% of cases) and bronchoscopy (48.7% of cases), the combination of CT scan and bronchoscopy was able to diagnose the etiology of the hemorrhage in 83.9% of cases [2]. However, there are still some rare causes that can result in a misleading clinical diagnosis, such as antiphospholipid antibody syndrome (APS) [3] and pseudoaneurysm [4]. Additionally, in some cases, the diagnostic procedures involving bronchoscopy were difficult and risky. Maxeiner [5] reported an unfortunate atelectasis patient who died from massive arterial bleeding after a bronchoscopic biopsy, in which a pathologic bronchial artery was violated by the biopsy procedure. Unless for airway control and isolation of the bleeding airway, for patients whose life-threatening hemoptysis has an unknown cause, bronchoscopy should be performed cautiously. Interventional angiography combined with BAE may be an alternative option. BAE can help in diagnosing and controlling the bleeding since 90% of massive hemoptysis cases emanate from the bronchial vasculature [1]. Miyano et al. [6] reported that, irrespective of the underlying causes, BAE could achieve up to a 93.3% successful treatment rate in massive hemoptysis management. Controlling the active hemorrhage may serve as a cornerstone for the subsequent management process for life-threatening hemoptysis.

ITA aneurysms are rare and commonly misdiagnosed as malignant tumors [7–9]. They usually occur in patients after sternotomy, endovascular procedures, trauma, chest wall infections, atherosclerosis, connective tissue diseases, or vasculitis [7–9]. Our patient was an otherwise healthy young female, and none of these underlying conditions were found. However, the limitation of our finding is the lack of histological examination for ruling out any possible causes, such as IgG4-related aneurysms, another extremely rare cause of ITA aneurysms [9].

Pregnancy has been reportedly associated with the development of aneurysms, presumably due to the associated hemodynamic and hormonal changes [10, 11]. However, ITA aneurysms in perinatal women have rarely been reported. Morimatsu et al. [10] reported a patient with life-threatening massive hemothorax due to the rupture of an ITA aneurysm after a vaginal delivery. However, there was no evidence linking the ITA aneurysm and the hemoptysis in this patient. However, given the rarity of such events and the considerable risk of producing misleading clinical diagnosis and therapeutic options, a detailed presentation of our findings might be helpful for life-threatening hemoptysis management. To the best of our knowledge, this is the first report of life-threatening hemoptysis concomitant with ITA aneurysms in perinatal women. BAE could be used as a first-line treatment irrespective of the underlying causes.

## Data Availability

All data generated or analyzed during this study are included in this published article.

## Abbreviations

FIB: Fibrinogen
TT: Thrombin time
PTR: Prethrombin time ratio
APTT: Activated partial thromboplastin time
PT: Prothrombin time
INR: International normalized ratio
MCV: Mean corpuscular volume
MCH: Mean corpuscular hemoglobin
MCHC: Mean corpuscular-hemoglobin concentration
CT: Computed tomography
BAE: Bronchial artery embolization
ITA: Internal thoracic artery
pro-BNP: Pro-B-type natriuretic peptide
APTT: Activated partial thromboplastin time
APS: Antiphospholipid antibody syndrome
